# Transcriptome Analysis of ESTs from a Chaetognath Reveals a Deep-Branching Clade of Retrovirus-Like Retrotransposons

**DOI:** 10.2174/1874357900802010044

**Published:** 2008-05-07

**Authors:** Roxane M Barthélémy, Jean-Paul Casanova, Eric Faure

**Affiliations:** LATP, CNRS-UMR 6632, Evolution biologique et modélisation, case 5, Université de Provence, Place Victor Hugo, 13331 Marseille cedex 3, France

**Keywords:** Retrovirus-like, retrotransposons, Chaetognatha, *Spadella cephaloptera*, reverse transcriptase.

## Abstract

Chaetognaths constitute a small marine phylum exhibiting several characteristic which are highly unusual in animal genomes, including two classes of both rRNA and protein ribosomal genes. As in this phylum presence of retrovirus-like elements has never been documented, analysis of a published expressed sequence tag (EST) collection of the chaetognath *Spadella cephaloptera* has been made. Twelve sequences representing transcript sections of reverse transcriptase domain of active retrotransposons were isolated from~11,000 ESTs. Five of them are originated from *Gypsy *retrovirus-like elements, whereas the other are transcripts from a *Bel-Pao* LTR-retrotransposon, a *Penelope*-like element and LINE retrotransposons. Moreover, a part of a putative integrase has also been found. Phylogenetic analyses suggest a deep-branching clade of the retrovirus-like elements, which is in agreement with the probably Cambrian origin of the phylum. Moreover, retrotransposons have not been found in telomeric-like transcripts which are probably constituted by both vertebrate and arthropod canonical repeats.

## INTRODUCTION

Chaetognaths are a small marine phylum living in various habitats, but most of them are among the most abundant planktonic organisms [1]. Their body is constituted of three parts, the head, trunk and tail, separated by septa [2]. These animals are protandric hermaphrodites; the ovaries lie in the trunk on both sides of the gut, while the testis are in the tail (reviewed in [3]). Their phylogenetic position remains enigmatic, although recent molecular analyses suggest a protostome affinity [4-7]. Casanova *et al.* [8] showed that chaetognaths can be considered as a model animal.

The chaetognath genomes exhibit several molecular singularities including paralog of both ribosomal RNA genes and ribosomal protein genes [9-11] and conservation of extremely divergent paralogous sequences suggesting a low rate of gene conversion [12]. Moreover, *in situ* hybridizations have shown that each type of both 18S and 28S rRNA paralogs is important for specific cellular functions [13-15]. The causes of these features are unknown, even if an alloploid event has been suggested [12]. As it is well known that mobile genetic elements (MGEs, also called transposable elements) can strongly impact genome evolution, knowledge of these elements about few is known in chaetognath [16] could be a fruitful contribution.

MGEs are ubiquitous in a wide range of living organisms; however, they make up a large fraction of genome sizes which is evident through the C-values of only pluricellular eukaryotes. These elements, which can transpose from one location to another within the genome, are known to be one of the causes of large scale genome reorganization [17]. Although regarded as a selfish DNA with negative impact on the host, MGEs have been shown to contribute significantly to gene evolution [18]. Now these elements are regarded as one of the principal forces driving the evolution of eukaryotic genomes [19,20]. Due to the great number of known MGEs (several thousands) and as new types of mobile repeats are discovered at a rapid rate, a unified classification system for eukaryotic transposable elements has recently been proposed, designed on the basis of the transposition mechanism, sequence similarities and structural relationships [21]. MGEs are divided into two classes. Class I retrotransposons replicate *via *an RNA intermediate; the key enzyme of this mechanism is the reverse transcriptase (RT), each complete replication cycle produces one new copy. Class II transposons, which are out of our topic, move as a DNA segment by a classical ‘‘cut-and-paste’’ mechanism.

Retrotransposons have been divided into five orders on the basis of their mechanistic features, organization and reverse transcriptase (RT) phylogeny: LTR-retrotransposons, *DIRS*-like elements, *Penelope*-like elements, LINEs and SINEs. The LTR-retrotransposons are retrovirus-like elements containing long terminal repeat (LTR) and ORFs for at least *gag*, a structural protein for virus-like particles, and *pol*. *Pol *encodes an aspartic proteinase (PR), reverse transcriptase, RNase H, and a DDE integrase (IN). LTR retrotransposons also contain specific signals for packaging, dimerization, reverse transcription and integration. The two main superfamilies *Gypsy *and *Copia*, differing in the relative order of integrase and RT domains. According to Wicker *et al.* [21], the other members are retrovirus, endogenous retroviruses (ERVs) and the *Bel–Pao* subfamily containing MGEs structurally similar to *Gypsy *or *Copia *elements but exhibit differences on RT phylogenies. Evolutionarily, LTR retrotransposons are closely related to retroviruses. Retroviruses have a viral lifestyle through acquisition of an envelope protein added to various regulatory proteins. Retroviruses are distributed widely among vertebrates and may also occur in some invertebrates, for example, some members of the *Gypsy *family found in dipteran insects are able to infect new individuals [22]. Retrovirus can also be transformed into an LTR retrotransposon-like through inactivation or deletion of the domains that enable extracellular mobility and can only be inherited by vertical transmission through the germ line, this is the case of the endogenous retroviruses even if some of them can always be transmitted horizontally.

*Penelope*-like elements (PLEs) represent a new order of retroelements identified in more than 80 species including unicellular animals, fungi and plants [23]. These elements code for a protein that represents a fusion between a reverse transcriptase and a GIY-YIG endonuclease. They encode an RT that is more closely related to telomerase than to the RT from LTR retrotransposons or LINEs; moreover, members of this order have also LTR-like sequences that can be in a direct or an inverse orientation. The principal other retrotransposon orders are the LINEs and the SINEs. The LINEs lack LTRs, can reach several kilobases in length, and are found in all eukaryotic kingdoms. Autonomous LINEs encode at least an RT and a nuclease in their *pol *ORF for transposition, they often display a poly(A) tail at their 3' end. The SINE elements are non-autonomous elements and as they do not contain RT gene, they rely on the activity of RT proteins encoded by LINEs to retrotranspose [24]. They originated from accidental retrotransposition of various polymerase III transcripts and possess an internal III promoter, allowing them to be expressed. SINEs belong to retrosequences, a group containing all the sequences arisen by reverse transcription of ribosomal, messenger and small stable RNAs [25,26].

A previous study using degenerate primers gave positive results during screening of *Sagitta *sp. for LINE-like and *Gypsy*-like reverse transcriptases, and also for *Mariner*-like transposases; however, only two LINE elements have been sequenced [16]. As in this phylum retrovirus-like sequences have never been documented, analysis of a published expressed sequence tag (EST) collection [6] of the chaetognath *Spadella cephaloptera* has been made.

## MATERIAL AND METHODS

### EST Sequence Identifications

The chaetognath EST collection used has been annoted by Marlétaz *et al.* [6]. Since then, a great number of new sequences have been deposited in databases. As a great level of sequence diversity could be found even in the same retrotransposon subfamily, this has necessitated a new analysis has been made. Since amino acid sequences are more useful to detect homology over long periods, the EST sequences were translated in all six reading frames and compared to the sequences in the NCBI nr and Swissprot protein databases. Sequences that did not match were further compared against the Gen-Bank and dbEST nucleotide databases (Blastn). Among 11,254 sequences, thirteen showed similarity (e-value<10^–5^) to previously described retroelement sequences.

### Blast and Phylogenetic Analyses

For each amino-acid sequences deduced from chaetognath ESTs which are homologous to retrotranspon genes are automatically searched for in the full length proteins from NCBI NR protein database. At this step, the Figenix platform has been used to automatically detect homologs based upon robust phylogenetic reconstruction [27]. When the number of homologs automatically detected is lower than 20, BLAST-based datasets were constructed using BLASTp queries against NCBI NR protein database. The Figenix platform has also been used for these phylogenetic reconstructions. The robustness of the tree has been tested by bootstrap analyses with 1000 resamplings. As for some analyses the number of homologous sequences was too low (< 8), only the alignments are given using the Clustal W program [28].

## RESULTS

### Blast and Phylogenetic Analyses

The cDNA library has been made from mRNAs isolated from various embryonic stages of *Spadella cephaloptera* (from 0 to 48 hours after hatching) [6]. The 5'-ends of 11,254 clones from this library have been sequenced and after annotation analyses the homology relations have been assigned to 2396 clones corresponding to the transcripts of 792 different genes. Similarly to Marlétaz *et al.*’s annotation [6], our re-analysis suggests that thirteen ESTs represent transcript sections of active retroelements (Table **1**). Three EST sequences are strictly identical and one sequence is internal to another EST. In spite the fact that alignments with published retrotransposons suggest that some EST sequences could partially overlap between them, it has been impossible to assemble these ESTs into contig due to significant nucleotide differences in the overlapping regions, suggesting that these ESTs belong to different MGEs although very close phylogenetically.

**Fig. (1):**
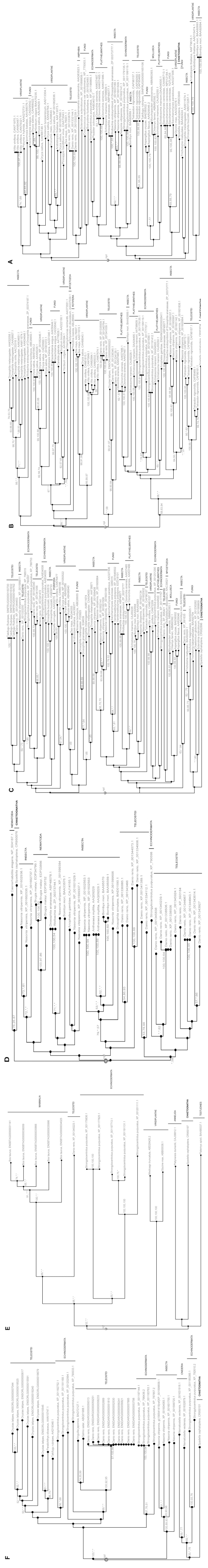
Phylogenetic trees from predicted translations of coding regions of reverse transcriptase domain of *Gypsy/Ty3* LTR-retrotransposons in (**A**), (**B**) and (**C**), of *Bel-Pao* LTR-retrotransposons in (**D**) and LINE elements in (**E**) and (**F**). The chaetognath EST accession numbers CR953949, (CR953634, CR953418, CR953554), CR952896, (CR950076, CR950075), CR950197 and CR950101 in respectively (**A**), (**B**), (**C**), (**D**), (**E**) and (**F**) phylogenetic analyses. Each tree is the fusion of three phylogenetic trees built on Neighbor Joining (NJ), Maximum Parsimony (MP), and Maximum Likelihood (ML) methods using FIGENIX software platform. For each node, bootstrap values are reported for each method. ^*^, means that the bootstrap value was inferior to 50% (e.g. a bootstrap 69,^*^,57 means that the node exists in NJ tree with a bootstrap value equal to 69, it exists in ML tree with a bootstrap value equal to 57, but does not exists in the MP tree). Branch lengths are correlated to the sequence evolution rate. Many of the same genes are mentioned more than once because they represent different GenBank sequences; since the trees are generated automatically, no discrimination was made.

Generally, in retrotransposons the *gag *and *pol *genes are intact; however some are interrupted by inframe stop codons, but some of the non-autonomous LTR-retrotransposons can transpose *via *trans-activation by autonomous partners. Only three of the chaetognath ESTs (CR950075, CR950076 and CR950197) do not contain stop codons; moreover, two ESTs have a microsatellite sequence linked to a part of the *pol *gene (Table **1**). In addition, in the chaetognath EST collection, only sequence homologies against Pol domain has been found; indeed, it is well known that the *pol *gene is the most conserved among the retrotransposons and to elucidate the phylogenetic relationships between these elements, *pol*-based trees have been constructed since Xiong and Eickbush [29]. Our analyses also shown that twelve out of thirteen of the ESTs encode part of the RT domain and generally the COOH-terminal amino acid region, whereas an EST sequence exhibits some homologies with a putative integrase (Table **1** and Fig. **1**).

Using Figenix platform, six phylogenetic trees have been obtained (Fig. **1**). Three analyses reveal that the deduced sequences of five ESTs belong to the *Gypsy/Ty3* superfamily (Fig. **1A**, **B** and **C**). These retrovirus-like elements have been found in animals, fungi and plants [21] and sequences belonging to these three taxa are present in the three phylogenies. Moreover, several clades of retrovirus-like elements have been found for the three taxa. Although the bootstrap values are very low, all these analyses suggest that the partial chaetognath sequences group with homologous regions of animal *Gypsy/Ty3 *elements. In two analyses, the chaetognath RT sequences group with fungi sequences or with fungi and plant sequences, but it is not statistically supported (Fig. **1A** and **C**), whereas in the other analysis, chaetognath sequence is the sister group of a a clade containing sequences from fish (Teleostei), echinoderms, plathyhelminthes and insectes (Fig. **1B**). In this analysis, chaetognath sequences seem to constitute a deep-branching clade of one of the animal retrovirus-like clade. In addition, another phylogenetic analysis suggests that the predicted sequence of two similar ESTs belongs to the *Bel-Pao* superfamily (Fig. **1D**) [30]; the chaetognath sequence appears to be the sister group of deuterostomian and plathyhelimnth sequences. In all the phylogenetic analyses using LTR-retrotransposons sequences, one or more bacterial sequences are included. They belong to the bacterial endosymbiont *Wolbachia *and exhibit a high homology percentage with the orthologs of their host (*Drosophila ananassae*); the bacterial sequence of the deduced *pol* gene of the *Gypsy *element and of the *Bel-Pao* element share respectively 703 and 1265 strictly contiguous identical amino acid with their insect orthologs. Similarly, in another phylogeny analysis, polymerase sequence of a Pineapple bacilliform virus (Retro-transcribing viruses, Caulimoviridae) [31]) groups with RT sequences from plants (Fig. **1A**). As it has been already shown for several other genes, this supports the hypothesis of horizontal gene transfers [32]. The two last phylogenetic analyses suggest that two chaetognath ESTs could encode part of the RT of a LINE element (Fig. **1E** and **F**). One predicted sequence group LINE elements from a turtle and an annelid, whereas the other groups with nematode and echinoderm sequences; however, in these two analyses the observed groupings are not statistically supported. Moreover, these phylogenetic analyses suggest that the chaetognath genome bears at least two types of LINE elements.

**Fig. (2):**
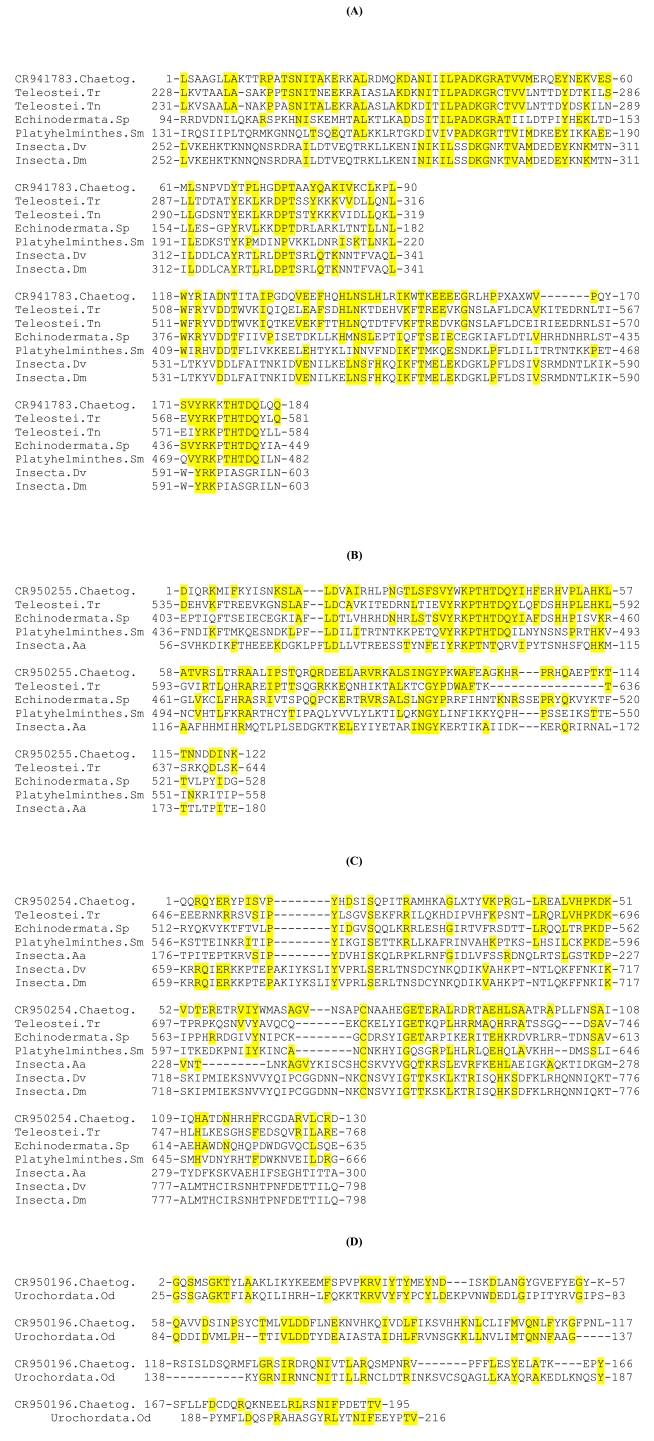
Alignments of predicted amino acid sequence of chaetognath ESTs with the homologous regions of various retrotransposons. (**A**), (**B**), (**C**) and (**D**) correspond respectively to alignments with CR941783, CR950255, CR950254, CR950196 chaetognath ESTs. In each alignment, the first sequence corresponds to the predicted amino acid sequence of a chaetognath EST. In **(A**), (**B**) and (**C**) the reverse transcriptase homologous sequences belong to Penelope-like elements, whereas in (**D**), only one putative integrase has been found partially homologous to chaetognath sequence. Sequences were aligned using program CLUSTAL W; positions of nucleotides are shown. The abbreviations of the other sequences and their accession numbers are: (Teleostei.Tr) *Takifugu rubripes*, AAK58879.1; (Telostei.tn) *Tetraodon nigroviridis*, CAF94542; (Echinodermata.Sp) *Strongylocentrotus purpuratus,* XP_001200565; (Platyhelminthes.Sm) *Schistosoma mansoni,* DAA00890; (Insecta.Aa) *Aedes aegypti,* AAZ15235; (Insecta.Dv) *Drosophila virilis* AAL14979; (Insecta.Dm) *Drosophila melanogaster,* AAR86938. Amino acid residues which are identical to those of the chaetognath sequences are underlined in yellow. Dashes represent gaps introduced to improve the sequence alignment. Stop codons appear as a result of unknown amino acid residue marked as X.

For the other chaetognath EST sequences, due to the low number of homologous sequences, phylogenetic analyses cannot be performed and the predicted amino acid sequences have been aligned with the homologous sequences given using Blast analyses (Fig. **2**). The deduced sequences from three ESTs exhibit homologies with parts of the RT domain of some of the *Penelope*-like elements (Fig. **2A**, **B** and **C**), whereas one EST shares partial homology with the COOH-terminal amino acid region of a putative integrase found in a urochordate (Fig. **2D**).

### Closed Relationships Between Retrotransposons and Microsatellites

Analysis of chaetognath ESTs reveals that two sequences contain part of retrotransposable elements stringly associated with the same microsatellite repeat motif (CAA)n (Table **2**). This motif has already been found in two out of the four microsatellite loci known of another chaetognath, *Sagittasetosa* (DQ463218 and DQ463220) [33]. However, in this last species, no MGEs have been found in the flanking sequences; the first loci is at the end of the ribosomal protein L8 gene, and the second in an intron of the Midasin gene (or pseudogene). Interestingly, several microsatellite families exhibit MGEs in their flanking sequences both in plants and animals [34-36] suggesting a possible close relationships between these two types of repetitive sequences.

### Research of Retrotransposons and Inverse Transcriptase Genes in Telomeric Regions

Telomeres are regions of repetitive DNA at the end of most of the linear chromosomes, which protects the end of the chromosome from destruction [37]. Telomerase, a ribonucleoprotein composed of a reverse transcriptase (RT) and an RNA encoded by a different gene, synthesizes the telomeric DNA repeats [38]. A relationship between telomerases and retrotransposon RTs, which are encoded by their template RNA, was surmised from shared amino acid motifs [38,39] and the observation that telomeres are elongated by non-LTR retrotransposons in some insects [40]. Indeed, as in almost all studied organisms, telomere repeats are very short simple sequences the exceptions are dipterans, which evolved chromosome ends with complex arrays of long satellite repeats in both *Chironomus* and *Anopheles gambiae* [41,42] or of non-LTR retrotransposons (members of *Drosophila *genus) that are elongated by telomerase-independent mechanisms (for review see [43,44]). *Drosophila melanogaster* telomeres are composed of two non-LTR retrotransposons, *HeT-A* and *TART*, with few copies of *Tahre*, an element that appears to combine parts of both *HeT-A* and *TART *and homologs of these two last elements have been found in *D. virilis *[45]. Moreover, one of the chaetognath EST sequences exhibits homology with the RT domain of the *Penelope*-like elements (Table **1** and Fig. **1D**). This order of retrotransposon has been found in animals, fungi and plants; in some taxa, they can be associated with telomeric repeats as shown by Gladyshev and Arkhipova [46]. According to these authors, they may descend from the missing link between early eukaryotic retroelements and present-day telomerases.

As a large number of ESTs have been found in telomeric regions of both in animals and plants (for example [47,48], consensus motifs of various taxa including animals, protozoa, plants and fungi [49] have been found in the chaetognath EST collection. ESTs containing telomeric-like sequences have been researched using all the known telomeric sequences. Only ESTs containing at least three telomeric motifs in a sequence of 153 nucleotides have been selected. Using this non stringent criterion, seventeen ESTs have been found and all but two contain both TTAGGG and TTAGG motifs (Table **2**). So (TTAGGG)n could be the ancestral telomere repeat motif of Metazoa. It has been conserved from the metazoan radiation in most animal phylogenetic lineages and according to our knowledge replaced by other motifs, only in two major lineages, Arthropoda [(TTAGG)n] and Nematoda [(TTAGGC)n] [50]. In the chaetognath EST collection, the mean nucleotide distance between two motifs is 15 nt and 19 motifs in tandem or more have been found. The nematode TTAGGC motif has very rarely been found but never in tandem or more, suggesting probably it is a TTAGG motif with a C as flanking base in 3’ end or a variant of the metazoan consensus.

Moreover, in addition to their role in protecting the ends of chromosomes, telomeres also influence the expression of adjacent genes, a process called telomere-position effect. The pattern of expression of the telomeric transgenes demonstrates that subtelomeric regions are epigenetically reprogrammed [51]. However, when the seventeen sequences were translated in all six reading frames and compared to the sequences in protein databases, the result has been negative indicating that these regions are not closely located to genes. A recent study suggests that in *Drosophila *an existing non-LTR retrotransposon was recruited to perform the cellular function of telomere maintenance [52]. However, in our analysis, the telomere flanking regions do not contain sequence homology with any retrotransposon element, and never even *Penelope*-like element, suggesting the presence of only consensus telomere repeats in chaetognath.

## DISCUSSION

Present analysis of a chaetognath EST library allowed to find thirteen retrotransposon sequences. Retrotransposons comprise 0.11% and 0.54 % of respectively the total ESTs and of the number of ESTs assigned to protein gene transcripts. In other animals, the corresponding values are generally higher, e.g., 1.0% in venom glands of a fish [53], from 4 to 14 according to the stages in a platyhelminth [54], and 14% in mouse oocytes [55]. However, the chaetognath value is similar to the 0.12% reported from the survey of ESTs from many plant species [56] but higher values could be found [57]. These transcriptional differences could be due to severe controls of the retrotransposon expression in higher plants, in which LTR-retrotransposons alone can comprise 50–90% of the genome [58] and probably also in juvenile chaetognaths. However, EST collection can exhibit several bias; indeed, ESTs are short single-pass sequence reads derived from cDNA clones selected randomly from cDNA libraries, and in contrast to genomic sequences, they are generally of low quality, poorly annotated, and highly redundant. Common EST features include ~2% sequence error rate with high frequency of insertions and deletions, redundancy of sequences derived from highly expressed genes, and low representation of genes expressed at low levels. Moreover, ESTs may derive from unspliced immature mRNAs, alternative splicing and polyadenylation sites, cloning artifacts (chimerisms), and mitochondrial transcripts [59]. In addition, it should be kept in mind that only transcription and ultimate integration in tissues giving rise to gametes is heritable and the chaetognath EST collection was derived from whole animals.

In spite the low number of ESTs encoding retrotransposons found in the chaetognath library studied, members of three out of four orders of autonomous retrotransposons have been found [21]; the lacking order is the one containing the *DIRS*-like elements. The higher number of transcripts has been found for the *Gypsy *retrovirus-like superfamily which could constitute a deep-branching clade according to phylogenetic analyses. The origin of chaetognaths remains obscure, but fossil evidences suggest that this phylum was widespread and diverse in the earliest Cambrian [60], and the difficulties of the phylogenetic position of this taxon is probably partly due to its divergence at an early stage from the primitive ancestor of the Bilateria. Moreover, as it is well known [61], present retrovirus-like phylogenies suggest the polyphyly of these elements.

In addition, one deduced amino acid sequence of two similar ESTs exhibits sequence homology RT of *Bel-Pao* LTR-retrotransposons issuing from deuterostomian and platyhelminthes. Moreover, phylogenies suggest the presence of active LINEs in the chaetognath genome; however, the level of homology between these sequences and the closer published sequences is very low. Phylogenetic analyses of LINE-like RT sequences suggested that sequences of the chaetognath *Sagitta* sp. group with those of Lophotrochozoa (i.e., Nemertea, Mollusca, Gastrotricha, Annelida, Echiura and Rotifera) [16], whereas in our phylogenies, the chaetognath LINE elements group with vertebrae and insect sequences.

Alignments of other predicted EST sequences with the closer orthologs obtained using Blast analyses show that the chaetognath genome also contains active elements which exhibit sequence homology with RT of *Penelope*-like elements issuing from deuterostomian, platyhelminthes and insects [39,62,63]. However, all our analyses show that the chaetognath retrotransposon sequences are not phylogenetically informative; this is principally due to the short size of the regions analyzed. Moreover, both in plants and animals numerous evidences of horizontal transmission have been published [64-66]; however, the basal positions of chaetognath sequences in most of our phylogenetic trees seem to exclude horizontal transfers.

Association between microsatellites and MGEs has been reported in a variety of organisms including plants and animals [34-36]. These MGEs could be DNA transposons, but many of them are retroelements. This results in microsatellites that have similar or nearly identical flanking regions that share great homology with MGEs, suggesting these last elements can be involved in the genesis and genomic spread of microsatellites in organisms as diverse as animals [34,35,67-69] and plants [70]. This intimate association between microsatellite repeats and retrotransposons has also been put to good use for develop a method named REMAP for genotyping and fingerprinting analyses [71]. The microsatellite–MGEs association could be a molecular symbiosis between two types of genomic sequences. Indeed, the two main molecular pathways of this mutual aid are by means of transposition and recombination. MGEs can not generally invade some chromosomic regions, due, e.g., to the absence of insertion sites or the presence of euchromatin. Moreover, MGEs can be strongly negatively regulated by various mechanisms, they even can encode their own negative *trans*-regulator [72, 73]. Recombination-related events due to microsatellites, such as unequal crossing over, allow the expansion of MGEs present in the microsatellite flanking regions, even when they are inactive. Contrarily, microsatellites flanking MGEs sequences could be multiplicated and dispersed in the genome during transposition processes. Transposition of additional regions including functional genes are known for both DNA transposons for which complex transposons probably evolve by transposition of homologous insertion sequences to nearby sites within a DNA molecule [74], and retrotransposons which can mediate sequence transduction [75]. Moreover, the 3’ end region of several non LTR-retrotransposons can be implicated as a major source for formation of adenine-rich microsatellites [67]. The potential molecular symbiosis between MGEs and microsatellites show that the behavior and the evolution of repetitive sequences can only be understood within a larger genomic context.

Our research of ESTs containing telomeric-like sequences reveals the presence of two types of telomeric motifs. The vertebrate motif (TTAGGG) is dominant; it constitutes an ancestral motif of telomeres in bilaterian animals and possibly also in the superclade including animals, fungi and amoebozoans [50,76]. More surprisingly, the probably ancestral Arthropoda motif (TTAGG)n [76] has also been found in ~30% of the cases, suggesting that it is not a type of degenerate TTAGGG repeats. Moreover, no sequence of retrotransposons or RT genes has been found in the telomeric regions.

The ability of various factors to stimulate MGE activity was first proposed by McClintock [77], and one of us (EF) has been involved in this field of research for a long time. With regard to LTR retrotransposons and retrovirus, chemical and physical agents have been shown to induce transcription and transposition [78-92]. Non-LTR retrotransposons also respond to these stress [93]. Moreover, expression of retrotransposon promoters are wound-inducible [94,95] and retrotransposons can be activated during viral, bacterial, fungal or parasitic attacks [96-98]. As retrotransposition burst can be an indicator of the stress genomic response, activation of MGEs will be investigated in chaetognaths, because this taxon seems very resistant. Indeed, chaetognaths do acquire eukaryotic and bacterial parasites but not very frequently [99,100]. There is no host-specific parasite known in chaetognaths, which is remarkable for such an old group [60]. Moreover, chaetognaths exhibit a great antibacterial activity [101] and in laboratory, no beheaded chaetognaths, which can survive some 30 days after decapitation and are able to mature spermatozoans and to mate with normal specimens; have exhibited bacterial infestation [102].

## CONCLUSION

In spite the low number of retrotransposon ESTs found in a juvenile chaetognath collection added to the small size of the sequences analyzed, this study suggests that chaetognath retrovirus-like retrotranposons could constitute deep-branching clades. The origin of these elements could correspond to the origin of the phylum; indeed, fossils of chaetognath grasping spines support the hypothesis that this animal taxon was present in the Cambrian times or even earlier. Moreover, studies on chaetognath retrotransposons could highlight future research in the exciting domain of the evolutionary origin of the retrovirus. Lastly, owing to the role of LTR-retrotransposons on genome structure, evolution and function, entire elements will be cloned and characterized, starting from the retrotransposon fragments shown in this study. Moreover, activation of these elements during normal development and in situations of stress, including pathogen attack will be sought.

## Figures and Tables

**Fig. (3):**
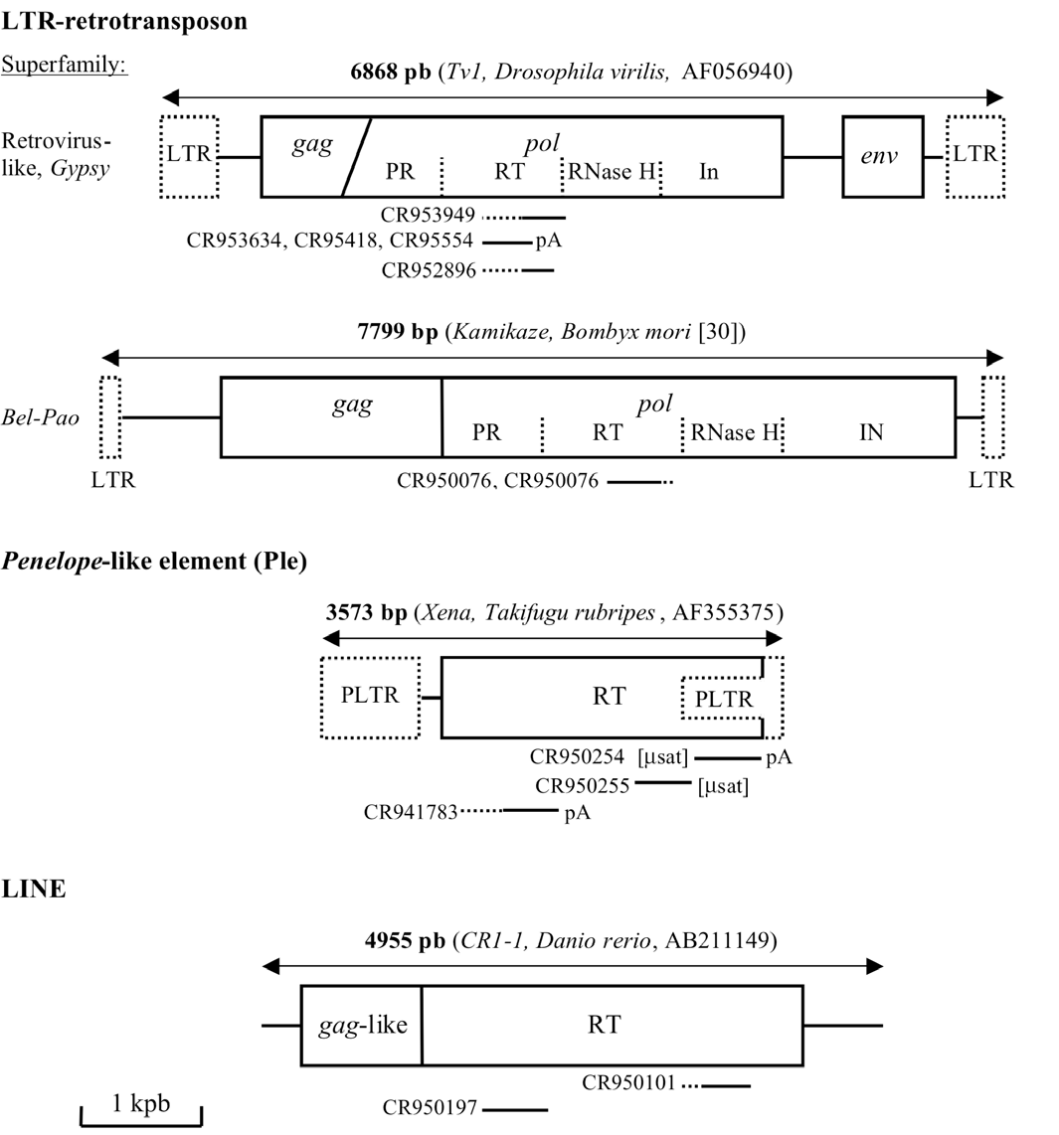
Mapping of the chaetognath ESTs bearing retrotransposon sequences on one of the closer complete retrotransposon genome. For each EST, homologous region between the retrotransposon is in continuous line, whereas, region which does not exhibit homology is in dash line. Concerning the retrotransposons, open 	reading frames are surrounded by continuous lines, non coding region are symbolized by a continuous line, whereas, long terminal repeats (LTR) or “pseudo-LTR” structure (PLTR) are surrounded by dash lines. For each retrotransposon, the length in base pairs (bp), its name, the name of the species bearing the element and the accession number or the corresponding reference are given. Abbreviations: [µsat] region containing microsatellite repeats, (pA) polyA trail, (*gag*) group antigen gene, (*pol*) polymerase gene, (*env*) envelope gene; and the various domains of the *pol* gene: (PR) protease, (RT) transcriptase inverse, (RNase H) RNase H, (IN) integrase.

**Table 1.** Chaetognath ESTs Containing Transcript Sections of Retrotransposon Domains

Type of Retrotransposon: Order and Superfamily for LTR- Retrotransposon	EST Acc. n°	Total Lenght - [Lenght of the Microsatelitte Region]- (Lenght of the poly(A) Tail)	Data Concerning the Closer Homologous Complete Retrotransposon: Name of the Element (if Known), Taxon, Species, Acc. n°	Protein Domain Matching	Other Caracteristic(s) of the ESTs
LTR-retrotransposon: Retrovirus-like, *Gypsy *	CR953949	641-(0)	*Tv1,* insect *Drosophila virilis* (AF056940)	RT-RNase H	One frameshift
LTR-retrotransposon: Retrovirus-like, *Gypsy *	CR953634 CR953418 CR953554	494-(23) 494-(23) 494-(23)	*Tv1,* insect *Drosophila virilis* (AF056940)	RT	The three sequences are stricly identical
LTR-retrotransposon: Retrovirus-like, *Gypsy *	CR952896	360-(0)	*Tv1,* insect *Drosophila virilis* (AF056940)	RT	
LTR-retrotransposon: *Bel-Pao*	CR950076 CR950075	784–(0) 682-(0)	*Kamikaze,* insect *Bombyx mori* [30]	RT	Shortest sequence internal to the longest
*Penelope*-like element (Ple)	CR950254	[53]-471-(26)	*Xena,* fish *Takifugu rubripes* (AAK58879)	RT	(CAA)n microsatellite
*Penelope*-like element (Ple)	CR950255	706-[303]-(0)	*Xena,* fish *Takifugu rubripes* (AAK58879)	RT	(CAA)n microsatellite
*Penelope*-like element (Ple)	CR941783	714-(30)	*Xena,* fish *Takifugu rubripes* (AAK58879)	RT	Complementary sequence
LINE	CR950197	574-(0)	*CR1,* testudine,* Platemys spixii* (AB005891)	RT	
LINE	CR950101	669-(0)	*CR1-1,* fish, *Danio rerio* (AB211149)	RT	
Putative integrase of an unknown element	CR950196	678-(30)	Urochordate *Oikopleura dioica *(AAS21408)	IN	Doubtful, homology with only one putative integrase

Several characteristics of each EST have given and of the closer homologous complete retrotransposon are reported. Abbreviations: (Acc. n°) Accession number, (RT) transcriptase inverse, (IN) integrase. The position of the number corresponding to the microsatellite sequence length reflects the position in the EST, i.e. in 5’ or in 3’ regions.

**Table 2:** Analysis of the Chaetognath ESTs Containing Telomeric Motifs

Number of Repeat	1	2	3	4	5	6	7
Motifs							
TTAGGG "Metazoan"	36	10	-	2	-	-	1
TTAGG "Arthropoda"	24	1	1	-	-	-	-
TTAGG/TTAGGG or TTAGGG/TTAGG	3	-	-	-	-	-	-
TTAGGG/TTAGGG/TTAGG	1	-	-	-	-	-	-
TTAGG/TTAGG/TTAGGG	1	-	-	-	-	-	-

The accession numbers of these ESTs are: CR940792, CR941840, CR944786, CR945111, CR945171, CR946582, CR946615, CR946633, CR946651, CR946666, CR947615, CR948211, CR949136, CR949498, CR949663, CR949907 and CR950054. - as 0.

## ABBREVIATIONS

MGE: = Mobile Genetic Element
DNA: = Deoxyribonucleic Acid
RNA: = Ribonucleic Acid
RT: = Reverse Transcriptase
LTR: = Long Terminal Repeat
Gag: = Group antigen
Pol: = Polymerase
PR: = Proteinase
IN: = integrase
PLE: = *Penelope*-like element
ORF: = Open Reading Frame
RNase H: = Ribonuclease H
ERV: = Endogenous Retrovirus
EST: = Expressed Sequence Tag
BP: = Bootstrap value
ENV: = Envelope
